# Supplementary material for: Label-Free Microfluidic Impedance Cytometry for Acrosome Integrity Assessment of Boar Spermatozoa
Source: Biosensors (Basel). 2022 Aug 25;12(9):679. doi: 10.3390/bios12090679 (PMC9496365; doi:10.3390/bios12090679)
Supplement: Supplementary file 1 [file biosensors-12-00679-s001.zip › biosensors-1842724-supplementary.pdf]

Supplementary information

Label-free microfluidic impedance cytometry for acrosome integrity assessment of boar spermatozoa

Stella A. Kruit[a,*], Douwe S. de Bruijn[a], Marleen L.W.J. Broekhuijse[b,c], Wouter Olthuis[a], Loes I. Segerink[a]

[a] *BIOS Lab on a Chip Group, MESA+ & TechMed Institutes, Max Planck Center for Complex Fluid Dynamics, University of Twente, 7500 AE Enschede, The Netherlands*

[b] *CRV Holding BV, 6843 NW Arnhem, The Netherlands*

[c] *Topigs Norsvin Research Center BV, 6641 SZ Beuningen, The Netherlands*

[*] *Correspondence:* s.a.kruit@utwente.nl *(S.A.K.)*

Supplementary figures

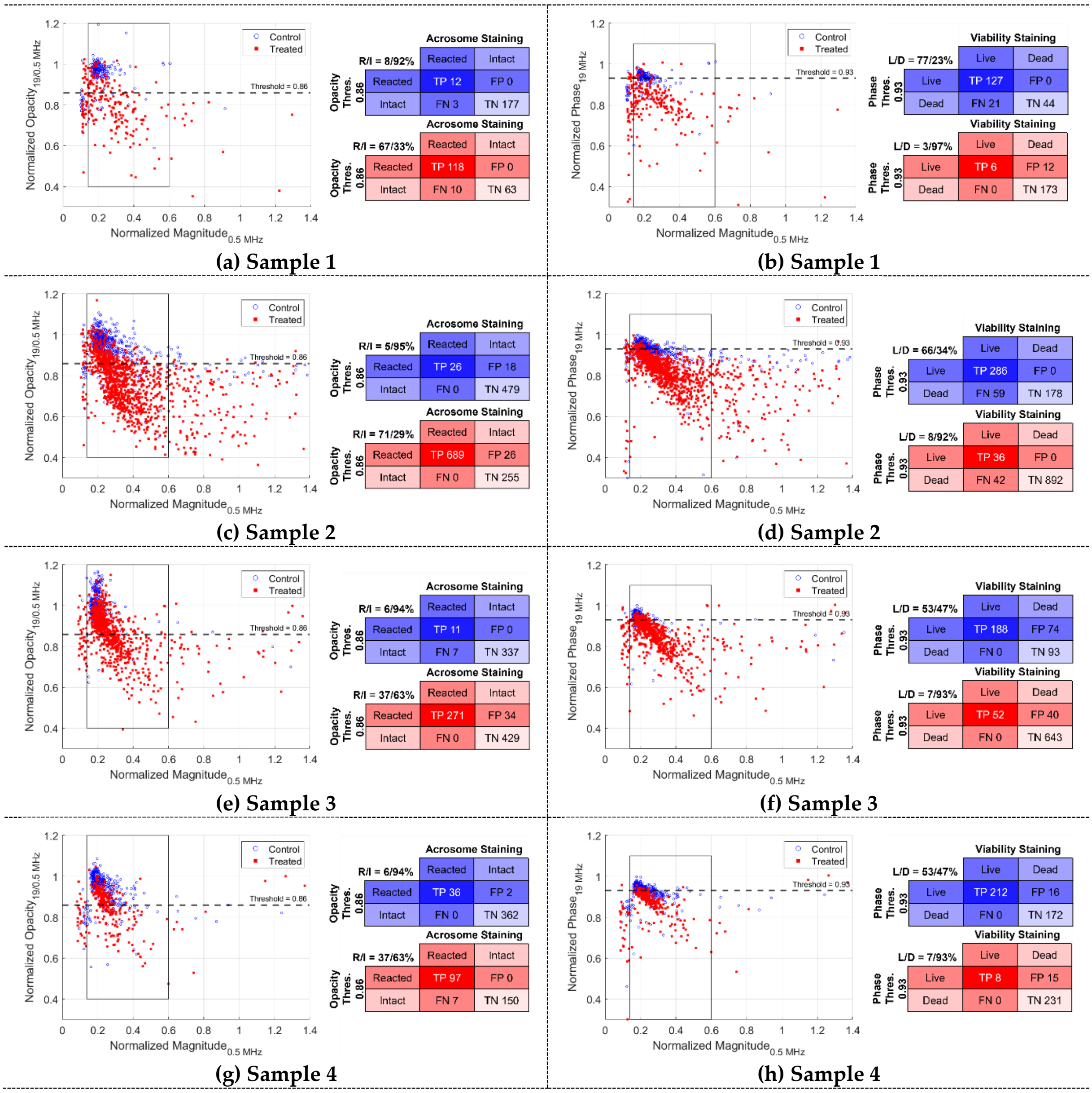

(a) Sample 1 **(b) Sample 1**
(c) Sample 2 **(d) Sample 2**
(e) Sample 3 **(f) Sample 3**
(g) Sample 4 **(h) Sample 4**

Figure S1: Scatterplots of normalized magnitude (0.5 MHz) vs. opacity (19/0.5 MHz) and normalized magnitude (0.5 MHz) vs. Phase (19 MHz) with their corresponding confusion matrix at a threshold of 0.86 and 0.93, respectively. The black square indicates the window of interest. a-b) sample 1. c-d) sample 2. e-f) sample 3. g-h) sample 4.

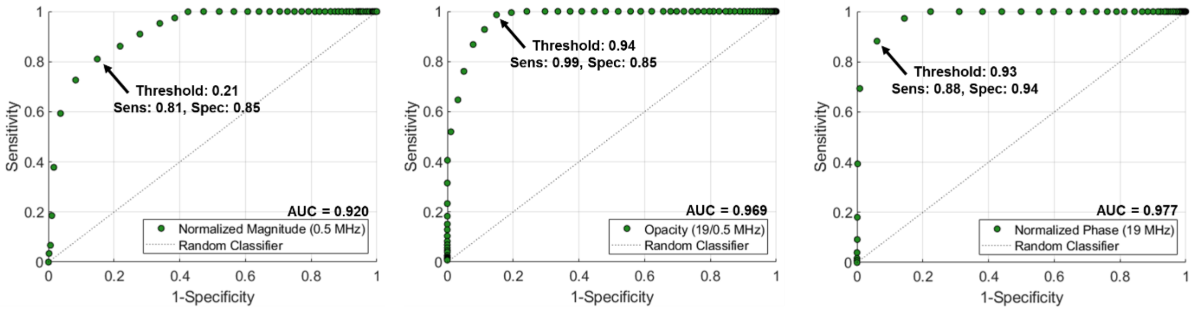

(a) **(b)** **(c)**

Figure S2: ROC curves to determine the sensitivity and specificity towards viability for (a) normalized magnitude (0.5 MHz), (b) phase (19 MHz) and (c) opacity (19/0.5 MHz). Phase at 19 MHz is found to be the most sensitive and specific for measuring viability.

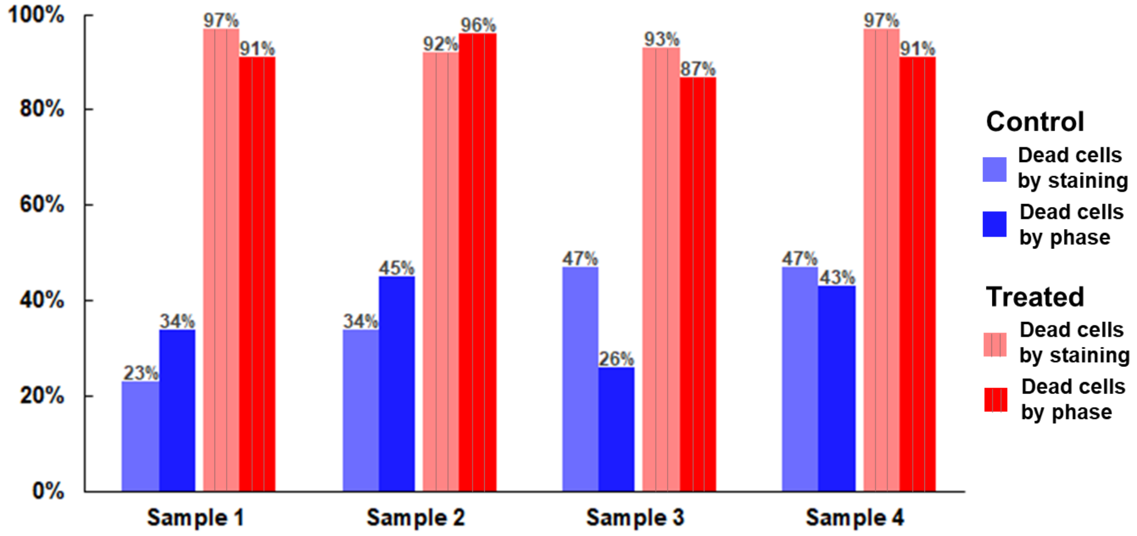

Figure S3: Determined viability by impedance phase at 19 MHz versus viability (PI positive) staining. A threshold of 0.93 is given as the compromise between sensitivity (88%) and specificity (94%) to give the best result for all control and treated samples.

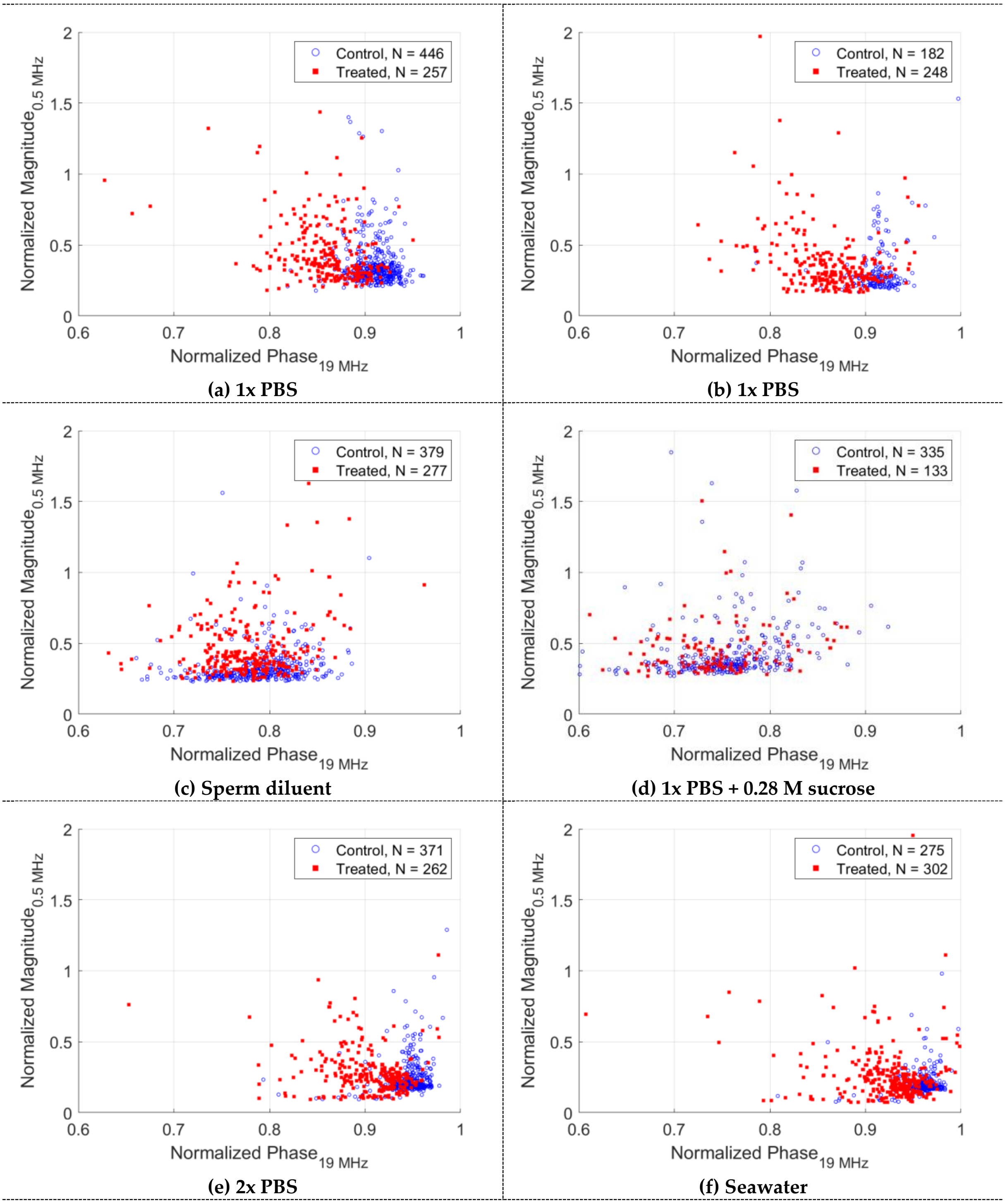

Figure S4: Scatterplots of normalized phase (19 MHz) vs. normalized magnitude (0.5 MHz) in buffers of varied conductivity and analyte composition. Impedance measured of control and treated sample in a-b) 1x PBS, c) sperm diluent (σ = 0.6 S/m), d) 1x PBS + 0.28 M sucrose (σ < 0.6 S/m), e) 2x PBS (1.6 < σ < 4 S/m), and f) seawater (σ = 4 S/m).
